# Progress in Medicine

**Published:** 1897-03-20

**Authors:** 


### Progress in Medicine.

### FEVERS.

(*Concluded from p. 398.*)

Sternberg[1] gave an important address on the etiology and classification of infectious diseases, in which he drew attention not only to the infective agent, but also to the predisposing factors, such as natural susceptibility, depressing agencies, local injuries, local congestion, and irritation forming a point of diminished resistance. With the exception of malaria and yellow fever, the infection is rarely spread through the atmosphere, or wafted across entire countries, which was formerly the accepted theory. There may be variations in the virulence of the infective virus, or in the susceptibility of the individual, or, lastly, a secondary infection of a different kind, any of which will modify the symptoms of the disease. The infective agent may come from outside, or may be a pre-existing parasite harmless in the ordinary conditions of itself and its host. Ziegler[2] denies that the processes set up in tissues after traumatism or infection are always beneficial; they are not necessarily purposeful—the individual does not always re-act in the way most beneficial to himself. Evolution has not adapted him to every new circumstance, the processes set up may be beneficial or harmful, and our efforts must be directed to distinguish between them. The subject of immunity is making rapid advances, and a brilliant account of its present position was given by W. B. Ransom at Nottingham.[3] He pointed out that it does not arise by exhaustion of the soil, for it occurs while the body fluids actually remain good culture media; nor by retention of bacterial products hindering their growth, for the same reason; nor entirely from phagocytosis, for the bacteria are at times destroyed by the fluids of the body before the phagocytes touch them. It has, indeed, been shown that eosinophilous cells approach anthrax bacilli, and discharge their granules at them. The latter quickly degenerate, and the true phagocytes then absorb and digest them. Even the action of the fluids may be either bactericidal or may only neutralise bacterial products. If it only neutralises their products, this may occasionally be by the production of a chemical antidote. More often all the cells of the body are stimulated to a protective reaction and become insensitive to the toxin, although we do not know that they secrete any chemical antidote. Thus antitoxic or protective serum, which in no sense neutralises a given toxin, will in the case of serpent venom, if injected, cause the body cells to protect themselves against a dose of the venom so quickly that it seems impossible for them to secrete a chemical antidote within the body. Just as we must distinguish all these different methods of defence, so it is to be noticed that immunity may be brought about by either a previous attack of the disease, by injection of a weak virus, and by increasing doses of virulent virus, by injections of two opposing microbes, or of bacterial products, or of serum from an immunised animal. Pfeiffer suggests that the protective substance exists beforehand in an inert state, like glycogen, in the liver, to be converted into an active form on due stimulation.

An interesting case of intra-uterine infection is discussed by Dürk.[4] A woman in the fourth week of typhoid gave birth to an infant who died in twelve hours. It was found that the spleen and liver were enlarged, and cultivations from them showed Eberth's bacillus and streptococcus albus, both of which were shown also in sections of the spleen. The bacilli must have penetrated some time before birth, since they were able to produce changes in the liver and spleen. The passage through the placenta is, he thinks, uncommon, and probably follows upon some injury to the cells covering the placental villi. Friedrich finds that the injection of sterilised cultivations of streptococci produces in man a fever which reaches its height in a few hours, while a second injection has less marked effects.[5] The filtrates have less effect than the sterilised cultures, showing that the proteids of the bodies of the bacteria have much to do in producing the result. Herpes facialis was produced by the injections in seven cases, and it was shown that the staphylococci found in some of the vesicles were not the cause of it. These observations are of some value in view of the numerous cases in which anti-streptococcal serum is being used. Thus, Williams has reported several cases of puerperal septicæmia, Ballance and Coleman others of poisoned hands in which it has been used with effect. Cook[6] narrates two pyæmic cases in which rapid decline of the symptoms followed its injection. The first was that of a labourer, aged 58, in whom inflammation of the leg and cellulitis occurred after a wound of the leg. The patient was doing very badly, with a temperature of 101 deg. and 102 deg. in spite of free incisions, but after injections of serum he made a rapid recovery. The other patient was a nurse who suffered from cellulitis after a finger prick. The result was more doubtful, as the patient was probably mending before the injection, but quickly completed her convalescence afterwards.

The treatment of the pyrexial condition cannot be determined by one invariable rule. Kast[7] showed that fever is not necessarily injurious, for many observers have found that animals survive infection better when kept at a high temperature, and the growth of the typhoid bacillus is, at any rate, not increased by pyrexia. Indeed, its power of multiplication is diminished 15 per cent. when fever is present; and neither phagocytosis nor leucocytosis are diminished, though how far the production of antitoxin is interfered with is unknown. The nervous system may, however, suffer from long-continued fever, and this is perhaps the chief reason for attempting to reduce it, though Von Jaksch lays stress on the formation of acids, and the consequent reduction of the alkalinity of the blood as an important effect of fever. Caution in the use of baths, guaiacol, saline injections, and other antipyretics is, therefore, recommended by a large number of writers. Hare,[8] while showing the advantages of cold baths over antipyretic drugs, argues that they must be modified to the case they are applied to in temperature and duration. Drury[9] notices the danger of guaiacol inunctions where there is cardiac weakness, though it acts with great regularity whatever is the disease. Claisse[10] describes the fall of temperature, general improvement, and diminished leucocytosis which follow saline injections. Lejar[11] gave injections of two or three quarts at a time, and once fourteen quarts in five days with great success, but lays down that the kidneys must be in a state to excrete the fluid. When they are not, Barre suggests his combined method of injection and bleeding.

**Small-pox.**—Matignon[12] brings forward a curious fact as to the short duration of variola immunity in China, where he found that at least 73 out of 122 vaccinations in children who had gone through small-pox three years previously were successful. He considers that Europeans in Pekin need to be vaccinated though they have suffered from small-pox, as the duration of the immunity is so short. Whether this arises from the climate, or from the existence of a special type of variola, is not explained. The anti-vaccinators in Germany are not satisfied with the practical annihilation of the disease, which now only destroys about four per million of the people. From the absence of danger they become bolder, and presented nearly 3,000 petitions against it in 1891. Drysdale refers[13] one benefit to their agitation, namely, the extended use of calf lymph. Prolonged consideration of the results of the Vaccination Commission endorses the value of the scientific conclusions embodied in it as apart from the recommendations based on policy; but nothing satisfies the opponents of vaccination, though the minority were unable to answer the facts contained in the report. The complete failure of improved sanitation to protect the unvaccinated, and the increased protection given where there are multiple vaccine marks, which are two beautiful applications of the canons of induction, remain unanswered. In the late campaign of the Congo State against the Arabs, Patterson[14] mentions, that of 25,000 persons in one camp, half of them suffered from the disease, while of some vaccinated soldiers only 1·26 per cent. were affected, and none died, though 50 per cent. of their comrades were attacked. In Hinde's book on the war he explains the great difficulty he had to get vaccine, while the Mohammedans were driven to save their slaves by inoculation, which, however, cost them a mortality of 50 per cent. The value of glycerine as a preservative for vaccine in temperate climes has been often referred to; W. G. King[15] now states that the use of lanoline is rapidly increasing in India for the same purpose, as it not only, like glycerine, prevents the growth of saprophytes, but also is unaffected by the climate. It is, however, chiefly used for the preservation of vaccine pulp as distinct from lymph, and has been known to preserve it uninjured for as much as two years and eight months.

The anti-vaccinators have already included in their crusade opposition to compulsory isolation, and, as the *Lancet* (October 31st) remarks, this suffices to show the disadvantage of the proposals of the minority on the Commission. Besides, with vaccination the individual can rely on his own care in seeing that he is vaccinated, whereas on a compulsory isolation system he is dependent on the honesty and efficiency of others in keeping infection from him. This, then, is the "more effective" method proposed in lieu of the invasion of the rights of the individual which vaccination entails. It has been also asserted that the unvaccinated children, who formed such a large proportion of those killed in modern epidemics, were the weakly ones who had been exempted from vaccination through their ill-health, but this cannot, as the *Lancet* (December 12th) notices, apply to Dewsbury, Leicester, and Gloucester, where only a small minority were vaccinated. Moreover, vaccination is said to be a great cause of ill-health, and yet the vaccinated children everywhere come out by far the best in epidemics. The Commission themselves point out that there was no difference in social rank or surroundings between the two bodies of children which exhibited this extraordinary difference. In fact, the amount of evidence for the reduction of mortality through vaccination and that alone, brought together by the Commission, is so vast that the reader is wearied of it. The *Lancet* goes on to urge that a national effort should be made to counteract the propaganda of the anti-vaccinators by condensing and distributing in the widest possible manner the evidence which proved to the Commission the efficacy of vaccination. The *British Medical Journal* urges that if the recommendations of the Commission are to be tried the trial must be temporary, and the mode of obtaining exemption must be made to exclude those persons who simply want to avoid the trouble of attending at a vaccination station. Thus the objectors should be compelled to go to obtain such exemption beforehand, and not be permitted to plead their conscience when summoned for neglect. The county councils, too, would be the fittest bodies for enforcing this very modified compulsion. The question of the storage and transmission of calf lymph is one that demands further investigation, and the journal thinks the payment and inspection of all private vaccinators to be unworkable. There is, however, some reason for the extension of the age for vaccination to six months, but a far more important matter is that re-vaccination should be made as compulsory as primary. Whatever other changes are made this should be pressed upon the Government which attempts to carry out the recommendations of the Commission.

[1] Amer. Jour. Med. Sciences, Dec. [2] Münch Med. Woch, Oct. 27. [3] Lancet, Dec. 19. [4] B. Med. Jour., Nov. 7. [5] Amer. Jour. Med. Sciences, Oct. [6] B. M. Jour., Oct. 31. [7] Amer. Jour. Med. Sciences, Oct. [8] Therap. Gazette, Oct. 15. [9] B. Med. Jour., Dec. 12. [10] Comtes Bendus de la Soc. de Biologie, 1896. [11] Therap. Gazette, Oct. 15. [12] Amer. Jour. Med. Sciences, Sept. [13] Med. Press, Sept. 9. [14] Lancet, Oct 29. [15] B. Med. Jour., Nov. 7.

